# The Structure of Glycerol Trinitrate Reductase NerA from *Agrobacterium radiobacter* Reveals the Molecular Reason for Nitro- and Ene-Reductase Activity in OYE Homologues

**DOI:** 10.1002/cbic.201300136

**Published:** 2013-04-18

**Authors:** Gustav Oberdorfer, Alexandra Binter, Silvia Wallner, Katharina Durchschein, Mélanie Hall, Kurt Faber, Peter Macheroux, Karl Gruber

**Affiliations:** [a] ACIB—Austrian Centre of Industrial BiotechnologyPetergasse 14, 8010 Graz (Austria); [b] Institute of Molecular Biosciences, University of GrazHumboldtstr. 50/3, 8010 Graz (Austria) E-mail: karl.gruber@uni-graz.at; [c] Institute of Biochemistry, Graz University of TechnologyPetersgasse 12, 8010 Graz (Austria); [d] Department of Chemistry, University of GrazHeinrichstr. 28/2, 8010 Graz (Austria)

**Keywords:** biocatalysis, crystal structures, oxidoreductases, OYEs, substrate binding, X-ray diffraction

## Abstract

In recent years, Old Yellow Enzymes (OYEs) and their homologues have found broad application in the efficient asymmetric hydrogenation of activated C=C bonds with high selectivities and yields. Members of this class of enzymes have been found in many different organisms and are rather diverse on the sequence level, with pairwise identities as low as 20 %, but they exhibit significant structural similarities with the adoption of a conserved (αβ)_8_-barrel fold. Some OYEs have been shown not only to reduce C=C double bonds, but also to be capable of reducing nitro groups in both saturated and unsaturated substrates. In order to understand this dual activity we determined and analyzed X-ray crystal structures of NerA from *Agrobacterium radiobacter*, both in its apo form and in complex with 4-hydroxybenzaldehyde and with 1-nitro-2-phenylpropene. These structures, together with spectroscopic studies of substrate binding to several OYEs, indicate that nitro-containing substrates can bind to OYEs in different binding modes, one of which leads to C=C double bond reduction and the other to nitro group reduction.

## Introduction

Ene-reductases from various organisms have been shown to catalyze the reduction of activated C=C bonds in α,β-unsaturated compounds efficiently.^1^–^16^ These enzymes are members of the Old Yellow Enzyme (OYE) family of proteins. In vivo, some OYEs are involved in the synthesis of important signal molecules in plant defense systems,^17^–^19^ whereas others are believed to participate in oxidative stress response.^20^ In general, however, there is only scant knowledge about the physiological roles of most OYEs.

**Scheme 1:**
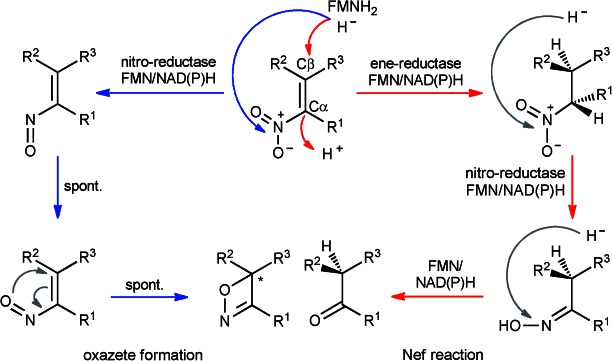
Reactions of nitro substrates catalyzed by OYEs. Blue arrows indicate the pathway that leads to the oxazete product. Red arrows show the reaction scheme leading to the carbonyl compound.

The asymmetric reduction of alkenes is an elegant and convenient way to obtain chiral compounds and was even honored with a Nobel prize.^21^, ^22^ In organic synthesis, the asymmetric reduction of such C=C bonds is usually facilitated by (transition-) metal catalysts.^21^, ^22^ Mechanistically, these catalysts perform the reduction in a *cis*-specific manner. In contrast, the biocatalytic analogue to this reaction is a *trans*-specific addition (Scheme 1).^1^, ^7^, ^12^, ^16^, ^23^

Structurally, OYEs have in common an (αβ)_8_- or TIM-barrel fold, conserved throughout the whole protein family. Characteristic of this fold is a central β-barrel that is closed at the bottom (N terminus) by a β-hairpin lid. Flavin-mononucleotide(FMN) is bound to these enzymes as a cofactor at the C-terminal edges of the β-strands.

In addition to the cofactor, the active site of such an enzyme typically consists of a His/His or His/Asn residue pair, which acts as H-bond donor to the electron-withdrawing group (e.g., a carbonyl, a carboxylic acid, or a nitro group) of a substrate molecule. In general, a conserved tyrosine residue in the active site acts as the proton donor during catalytic turnover.^1^, ^7^, ^23^, ^24^ It has been shown, however, that in some OYEs (such as pentaerythritol tetranitrate reductase, PETNR) this residue is not essential for catalysis and that its role can be taken by a water molecule.^15^

The exact shape of the substrate and cofactor binding site is determined by loops connecting the central β-barrel strands with the subsequent α-helices. These loops (subsequently termed β1 to β8, Figure S1 in the Supporting Information) exhibit significant sequential and structural diversity among OYEs from various organisms.^25^ We have recently correlated structural features and sequence motifs of OYEs with their stereopreferences in the reduction of nitro-olefins; this has allowed a classification of these enzymes, as well as the prediction of their catalytic properties based on their amino acid sequences.^26^

Some members of the OYE family were originally isolated from bacterial strains that are able to use nitroglycerin (glycerol trinitrate, GTN) as their sole nitrogen source^27^, ^28^ by removing either one or two nitrate groups from GTN. The enzymes PETNR from *Enterobacter cloacae*,^29^ xenobiotic reductases XenA and XenB from *Pseudomonas putida* and *Pseudomonas fluorescens*,^30^ NerA from *Agrobacterium radiobacter*, and OYE1 from *Saccharomyces pastorianus* have been shown to catalyze the removal of the first nitro group of GTN through a reductive denitration mechanism, releasing nitrite. NerA has been biochemically described in greater detail^31^–^33^ and was also shown to reduce a number of α,β-unsaturated compounds.^34^ Recently, it has been shown that NerA and other ene-reductases convert nitroalkenes into highly strained oxazetes (Scheme 1) and are also capable of catalyzing a biocatalytic analogue of the Nef reaction.^34^, ^35^

The exact mechanism of oxime production is still a matter of debate, however, and it has also been reported to proceed via the alkene substrate.^36^ The disparity between these two observations has not been fully clarified and might be due to differences in reaction conditions. Careful analysis of the literature thus revealed that NerA, as well as other OYE homologues (including XenA, morphinone reductase, OPR3, and PETNR), show dual activity on a range of nitro-olefins, combining both C=C bond reduction and nitro reduction. More precisely, NerA showed exclusive nitro reduction in the case of an extended conjugated system (e.g., with 1-nitro-2-naphthylpropene), whereas C=C bond reduction strongly predominated in a poorly conjugated system (e.g., with nitrocyclohexene). Intermediate cases were observed with substrates bearing a phenyl substituent at Cβ, with comparable levels of competing ene-reductase and nitro-reductase activities being determined (alkyl substitution at Cα almost completely inhibited nitro-reductase activity). Understanding the preferred reaction pathway catalyzed by OYE homologues is crucial, because it would provide a model to direct the conversion of nitro-olefins either towards the industrially relevant asymmetric synthesis of nitroalkanes or towards the environmentally important detoxification of nitro pollutants.

Here we present the X-ray crystal structure of glycerol trinitrate reductase (NerA) from *A. radiobacter*. We determined structures of this enzyme in its apo form, and also after soaking either with the OYE-typical inhibitor 4-hydroxybenzaldehyde or with 1-nitro-2-phenylpropene, the model substrate for the nitro-reduction reaction. The structures confirm the enzyme to be a member of the OYE family and verify activity predictions from an analysis of sequence patterns.^26^ Furthermore, the observed binding modes both for the inhibitor and for the substrate provide a direct explanation for the observed ene-reductase activity of this enzyme. Through a comparison of the active site structure of NerA with those of other OYE-like enzymes and UV/visible spectrophotometric measurements we were able to identify the structural differences that might account for nitro-reductase and ene-reductase activities observed with particular OYEs.

## Results and Discussion

### Structure of NerA

**Figure 1:**
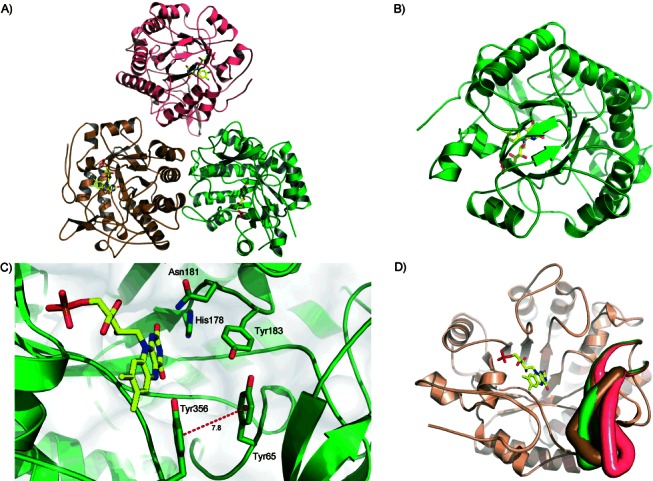
Crystal structure of NerA. A) Three monomers are present in the asymmetric unit of the NerA structure. Each monomer is shown in a cartoon representation (chain A brown, chain B green, and chain C salmon) with FMN displayed as sticks and colored in yellow. B) NerA viewed from the bottom of the β-barrel, showing the β-hairpin structure typical for OYEs. C) Active site of NerA. The active site residues participating in catalysis are shown as sticks and labeled according to NerA sequence numbering. Pseudoatom centers are placed in the middles of the aromatic rings of Y65 and Y356.^26^ D) Superposition of all three NerA monomers present in the asymmetric unit in a *B*-factor putty representation. The loops are colored according to the three different chains (chain A brown, chain B green, and chain C salmon). The figure was prepared with PyMOL.

The protein was purified by affinity and size exclusion chromatography as described previously,^34^ and the purified protein was used for crystallization trials. The X-ray crystal structure of NerA was determined from an orthorhombic crystal (space group *P*2_1_2_1_2_1_) to a resolution of 1.6 Å by molecular replacement using the structure of PETNR (PDB ID: 1h50) as a search model. Three enzyme molecules were present in the asymmetric unit (Figure 1 A), and the final model could be refined to *R* and *R*_free_ values of 15.8 and 18.9 %, respectively (Table 1). By rules that correlate oligomeric states and active site shapes of OYEs with specific sequence patterns,^26^ NerA had been predicted to be monomeric. An analysis of interchain contacts within the crystal with the aid of the PISA server^37^ indeed found no evidence for oligomerization in solution, and this was also confirmed by size exclusion chromatography (Figure S2). The size of NerA in solution was determined by analytical size exclusion chromatography. By use of a calibration curve created with the LMW Gel Filtration Calibration kit (GE Healthcare) the molecular weight of NerA was determined to be approximately 40 kDa, which corresponds to the monomeric form of the protein.

**Table 1:** Diffraction data and refinement statistics[a]

	Native NerA	Complex with	
		4-hydroxybenzaldehyde	1-nitro-2-phenylpropene
**Data collection**			
X-ray source	ESRF, ID23-1	SLS, PX-III	SLS, PX-III
*λ* [Å]	0.9724	1.00	1.00
*T* [K]	100	100	100
space group	*P*2_1_2_1_2_1_	*P*2_1_2_1_2_1_	*P*2_1_2_1_2_1_
*a*, *b*, *c* [Å]	68.45, 93.02, 180.50	58.91, 68.28, 90.06	60.15, 69.98, 91.99
resolution [Å]	90.25–1.60	45.03–2.29	50.34–2.49
	(1.69–1.60)	(2.41–2.29)	(2.62–2.49)
total no. reflections	469 303	31 200	26 825
unique reflections	149 769	17 038	14 125
multiplicity	3.1 (2.9)	3.7 (3.6)	3.5 (3.5)
completeness [%]	99.1 (97.5)	98.0 (93.1)	99.9 (99.8)
*R*_p.i.m._	0.071 (0.290)	0.071 (0.184)	0.123 (0.419)
*R*_sym_	0.107 (0.413)	0.123 (0.309)	0.200 (0.677)
<*I*/*σ*_I_>	7.00 (2.3)	7.4 (3.6)	4.2 (1.5)
**Refinement**			
resolution [Å]	38.5–1.60	45.0–2.29	46.0–2.49
*R*_work_/*R*_free_	0.1587/0.1895	0.1943/0.2391	0.2135/0.2625
**No. of atoms**			
protein	8706	2822	2795
cofactor/substrate	93	40	51
water	1868	218	96
***B*** **factors [Å^2^]**			
protein	16.4	17.9	26.8
cofactor/substrate	5.7	14.9	29.4
water	26.3	19.3	23.9
all atoms	17.9	18.0	26.8
**RMSD**			
bond lengths [Å]	0.006	0.007	0.007
bond angles [°]	1.07	0.96	0.90

[a] Values in parenthesis relate to the highest-resolution shell.

The overall fold of NerA is a TIM barrel, with the typical OYE N-terminal (residues 2–18) β-hairpin lid closing the bottom of the central eight-stranded parallel β-barrel structure (Figure 1 B). Strands and helices are connected by loops, which contain the essential catalytic residues as well as residues building up the substrate and cofactor binding sites (Figure 1 B). The binding site of the FMN cofactor is found at the C-terminal edge of the parallel β-strands (Figure 1 C). Residues stabilizing the electron-withdrawing group (EWG; e.g., carbonyl, carboxylic acid, nitro) of a ligand in an ene-reductase-like reaction are a histidine/asparagine pair (H178 and N181). The putative proton donor residue is Y183. However, it is known from other ene-reductases that water molecules can also facilitate proton transfer.

Structural superposition of the three monomers in the asymmetric unit gave very low RMSD values (Cα-RMSD for 371 aligned residues) of 0.160 Å for the superposition of chain A and chain B, 0.097 Å for that of chain A and chain C, and 0.216 Å for that of chain B and chain C. Larger structural differences are found in the regions of loop β3 (Figure 1 D). The loops connecting β strands 5 and 6 with their subsequent α-helices are rather shorter in NerA than in the molecular replacement template, PETNR (and other OYE-like proteins). In contrast, loop β3 in NerA is very long and forms a small subdomain. Loops of comparable length have only been described for two other OYE like proteins—PETNR from *E. cloacae* and SYE-1 from *Shewanella oneidensis*.^15^, ^25^, ^38^ Similarly to these two structures, NerA exhibits the highest crystallographic *B* factors in this capping subdomain (Figure 1 D).

### Classification through structural clusters and sequence patterns

To characterize the crystal structure of NerA further, we analyzed how it fits into a set of structure-based activity relationships recently identified for OYEs.^26^ These relationships are based on the distance between two (most often aromatic) residues in the active site, which are important for substrate binding and suitable orientation for the *trans*-hydrogenation reaction (Scheme 1). In the case of NerA, these residues are Y65 and Y356, and the geometric centers of their phenyl rings are 7.8 Å apart from each other (Figure 1 C). This distance places NerA in the “intermediate distance cluster” of OYEs.^26^ Members of this structural cluster were found to exhibit only moderate stereoselectivity in the reduction of 1-nitro-2-phenylpropene. Structures belonging to this cluster are also prone to alteration of binding modes, depending on the reaction conditions and/or the cofactor recycling system.^8^, ^26^ The experimentally determined moderate stereoselectivity of NerA with 1-nitro-2-phenylpropene (54 % *ee* for the reduced *S*-configured product)^34^ is in line with this prediction. Moreover, the sequence of NerA also contains the motifs GYADVPGLY around loop β2 and PLTRNR around loop β1 (see Figure S1 for a description of the loops). These have previously been shown to correspond to the observed structural features: that is, the distance between the two aromatic residues and the oligomeric state of the enzyme (see above).^26^

### NerA complex structures

The structures of NerA in complexation with 4-hydroxybenzaldehyde and with 1-nitro-2-phenylpropene were determined at 2.3 and 2.5 Å resolutions from orthorhombic crystals (space group *P*2_1_2_1_2_1_). The previously determined apo structure was used as the search model for molecular replacement. Both soaking structures had one molecule present in the asymmetric unit. The overall *B* factors were higher than in the apo structure, and the overall structures were identical to the apo one. Like in the apo structure, weaker density was observed for the loop β3 region in both complex structures.

**Figure 2:**
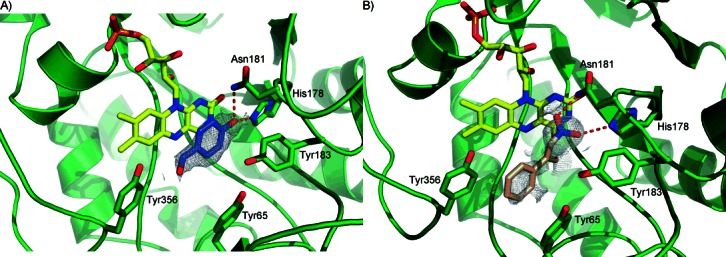
Substrate and inhibitor complexes of NerA. A) Close-up view of the structure of NerA in complexation with 4-hydroxybenzaldehyde. B) Close-up view of the NerA structure in complexation with 1-nitro-2-phenylpropene. In both cases, the 2 *mF*_o_−*DF*_c_ electron density map before the ligands were fit into the density, countered at 1*σ* are shown. Important active site residues are shown as sticks and labeled by NerA sequence numbering. The figure was prepared with PyMOL.

The electron density for the soaked inhibitor 4-hydroxybenzaldehyde allowed fitting of the compound with the aromatic ring placed parallel to the *si*-face of the FMN isoalloxazine ring and the hydroxy moiety bound between residues H178 and N181 (Figure 2 A). The aldehyde moiety, on the other hand, was fitted in the space between the aromatic residues Y65 and Y356. Such a binding mode is typical for this inhibitor when bound to OYEs^39^ and conclusively provides experimental evidence that NerA binds carbonyl ligands in an OYE-like fashion.

The density for the soaked substrate 1-nitro-2-phenylpropene was only weak and was interpreted in a way that corroborates NerA's ability to bind substrates in an OYE-like fashion. Like the 4-hydroxybenzaldehyde, 1-nitro-2-phenylpropene binds in an orientation that would facilitate ene-reductase activity (Figure 2 B). During the refinement, the occupancy and mean *B* factor of 1-nitro-2-phenylpropene were refined to 0.92 and 40.59. In the observed binding mode, the nitro group is stabilized through H-bonding to residues H178 and N181. The C=C double bond is oriented in ideal geometry and distance for proton donation (3.5 Å) from Y183 onto Cα, while at the same time Cβ is in hydride transfer distance (3.8 Å) from N5 of the isoalloxazine ring. The aromatic ring is bound between the tyrosine residues Y65 and Y356 through stacking interactions. This conformation leads to formation of the *S* enantiomer, consistently with the experimentally determined stereospecificity of the enzyme.^34^

This binding mode, however, deviates substantially from others obtained by soaking a relatively similar substrate—2-[(*E*)-2-nitrovinyl]phenol—into crystals of PETNR^36^ (PDB IDs: 3p80, 3p81, 3p7y). A comparison between these structures and that under discussion here made it clear that, in the absence of the additional hydroxy group from the substrate used by Toogood et al., the nitro group is stabilized between residues H178 and N181. This is further corroborated by two other structures present in the PDB (PDB IDs: 4eab and 4ab4) of the ene-reductase XenB in complexation with TNT. In these two structures, one of the three nitro groups of the substrate is always stabilized between residues corresponding to H178 and N181 in NerA, in a binding mode similar to that observed here.

### Ene-reductase versus nitro-reductase activity

In addition to their capability to hydrogenate activated C=C bonds in α,β-unsaturated compounds, some OYEs (including NerA) have been shown to catalyze nitro-reduction reactions in both saturated (through a biochemical analogue of the Nef reaction)^34^ and unsaturated nitro compounds (forming the highly strained oxazete system as an electrophilic cyclization product).^35^ In the case of a substrate bearing a nitro functionality as the EWG the reaction is facilitated by hydrogen bonds, similarly to other EWGs, but hydride transfer and protonation are not necessarily concerted. Instead, a nitronate species might be formed after the hydride has been transferred from the reduced flavin to the Cβ-position of the substrate. This intermediate has a much lower binding affinity and might dissociate. However, protonation is then facilitated again by an active site tyrosine residue at the Cα-position of the substrate.^40^ It should be noted that some OYEs (such as PETNR) have been shown to catalyze the reduction of 1-nitro-2-phenyl-like substrates without the formation of nitronate intermediates.^15^

In the case of nitro reduction, the hydride has to be transferred directly onto the nitrogen atom. Thus, in OYEs capable of producing either the carbonyl compound (Nef reaction analogue) or the oxazete product, a second, competing binding mode of the substrate to the enzyme must exist. It was therefore anticipated that the soaked substrate 1-nitro-2-phenylpropene would bind in two different ways to NerA. However, no ambiguous density interpretable as a second binding mode was detected. Instead, density for a binding mode that would result in the reduction of the C=C double bond was found (Figure 2 B). It is thus not clear from the X-ray complex structure alone why NerA is capable of reducing either a C=C double bond or a nitro group. To investigate the reason for this dual activity further, a comparison of the active site cavities of OYEs reported to catalyze nitro reduction^34^, ^35^ (XenA, OPR-3, PETNR, and NerA) was performed. Subsequently, the active sites of OYEs with nitro-reductase activity higher than ene-reductase activity were compared with the active site cavity of OYE-1, which is strictly selective for C=C reduction.

**Figure 3:**
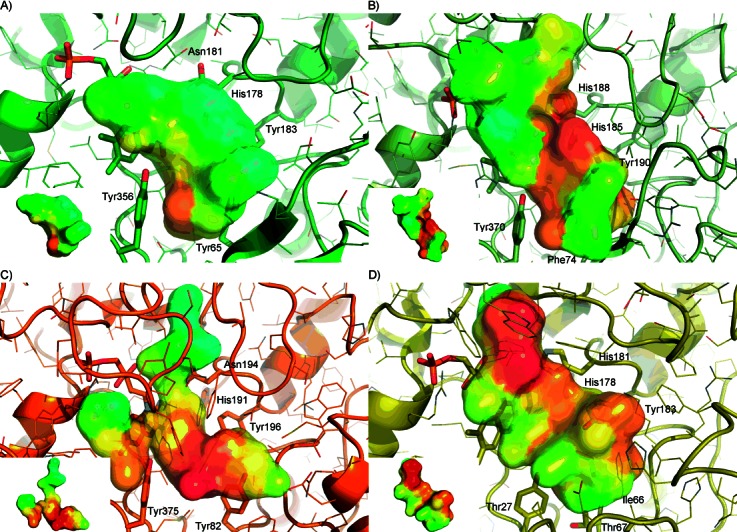
Active sites of A) NerA, B) OPR-3, C) OYE-1, and D) XenA. The colored, semi-transparent surfaces each represent the shape of the associated enzyme's active site cavity. The surfaces of the cavities are colored according to the hydrophobicity/hydrophilicity of the residues lining the active site (red hydrophobic, blue hydrophilic). The small insets in the lower left corners each show a cavity representation without the surrounding enzyme. The figure was prepared with PyMOL.

The qualitative analysis was started by comparison of the sizes and shapes of the enzyme active site cavities, but this resulted in no obvious or characteristic differences between OYEs with pronounced nitro reducing activity or C=C bond reducing activity. This finding made it clear that size and shape are not exclusive determinants of the course of the reaction. Nonetheless, the comparison revealed that the sizes and shapes of OYE active sites are very diverse (Figure 3 A–D). This was unexpected because the proteins are similar in terms of overall size, shape, and number of amino acids, with a considerable degree of sequence conservation (≍30 to <80 %).

To gain deeper insights into the differences of OYEs' active sites, we analyzed the hydrophobicity/hydrophilicity distributions in the cavities (Figure 3). For NerA we found that the biggest part of its active site is hydrophilic, with only a small hydrophobic patch located between the residues Y65 and Y356 (Figure 3 A). In addition, a relatively hydrophobic region can be found parallel to the isoalloxazine ring of FMN; this indicates favorable binding of the substrate's aromatic/hydrophobic part in an orientation that results in a C=C double bond reduction. This is in line with the experimentally determined activity of NerA for the model substrate 1-nitro-2-phenylpropene^35^ and with the complex structure (Figure 2 B). However, NerA was also shown to yield reduction products of the nitro group to a lesser extent (overall nitro-reductase/ene-reductase activity ratio 1.9). The biocatalytic performance and active site structure of NerA are comparable with those of OPR3 (Figure 3 B), an enzyme also known to catalyze both reactions (nitro-reductase/ene-reductase activity ratio 1.2).^34^, ^35^ Both enzymes have hydrophobic patches in their active sites, but this hydrophobicity is in each case more evenly distributed throughout the whole cavity rather than focused on a single spot (Figure 3 A and B).

Upon mapping hydrophobicity onto the active site of OYE-1 (Figure 3 C), which was shown to exhibit exclusive ene-reductase activity,^34^ a defined hydrophobic “hot-spot” was found between two active site tyrosine residues (Y82 and Y375). These two residues had previously been identified to be important for substrate binding (accommodating the hydrophobic phenyl ring of the substrate) and stereoselectivity of OYE enzymes.^26^ A “hydrophobic hot-spot” between these residues favors a binding mode in which the nitro group of a substrate is stabilized by H-bonds to the active site histidine/asparagine pair. In such a binding mode the C=C bond is placed parallel to the plane of the FMN cofactor isoalloxazine ring, thus resulting in a geometry favorable for alkene reduction.

The reverse conclusion dictates that an OYE enzyme furnishing mostly oxazete and acetophenone (final nitro reduction products, Scheme 1) has to have a “hydrophobic hot-spot” in an area of the active site that favors a binding mode in which the nitro group is placed in a geometry and with a distance between N5 and FMN that are ideal for hydride transfer. This is indeed observed in the case of XenA (Figure 3 D). In the X-ray crystallographic structure of this enzyme, a “hydrophobic hot-spot” is formed by the upper loops β5 and β6 rather than the lower loops (β2 and C term, Figure S1); this provides the ideal chemical environment for binding the hydrophobic/aromatic tail of the tested substrate (Figure 3 D). Biotransformations with XenA show a stronger preference—by one order of magnitude—for nitro-reductase activity with 1-nitro-2-phenylpropene. In fact, measuring the distance from the end of cavity loop β6 (Figure S1) to a pseudoatom placed between N5 of the flavin cofactor and the OH group of the active site tyrosine residue yields a value of about 7 Å: the length of the 1-nitro-2-phenylpropene substrate. Placement of the nitrogen atom of the NO_2_ group at the calculated pseudoatom position would result in H-bonds formed between both nitro oxygen atoms to active site tyrosine residues.

### Spectroscopic studies

**Figure 4:**
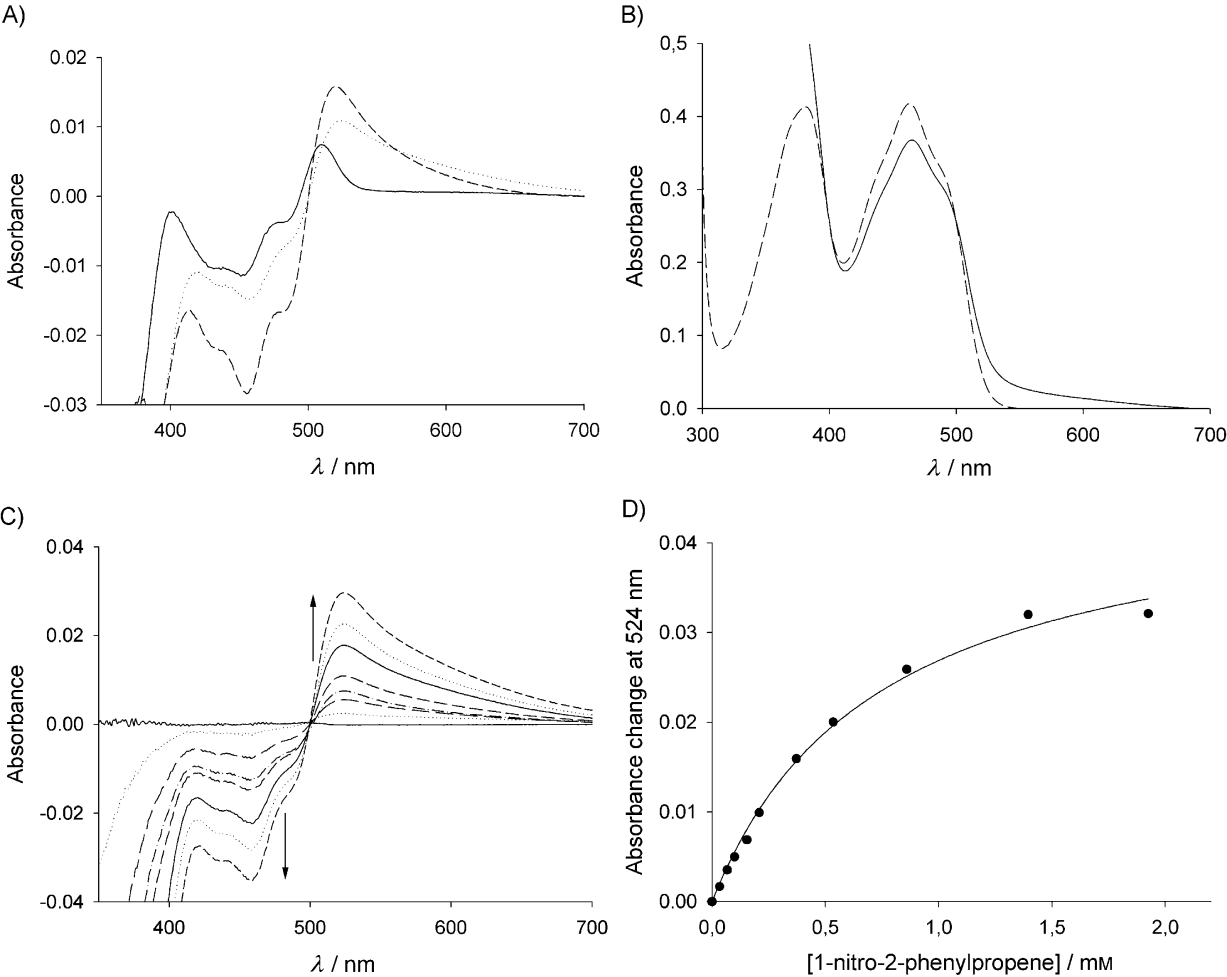
UV/Vis spectroscopy. A) UV/Vis absorbance difference spectra at concentrations of 1-nitro-2-phenylpropene corresponding to the *K*_d_ values listed. Solid line: OYE1 (*K*_d_=0.17 mm). Dotted line: NerA (*K*_d_=0.73 mm). Dashed line: XenA (*K*_d_=0.085 mm). B) UV/Vis absorbance spectrum of NerA before (dashed line) and after (solid line) titration with 1-nitro-2-phenylpropene. C) Difference spectra of NerA at increasing concentrations of 1-nitro-2-phenylpropene. D) Determination of the *K*_d_ value for the binding of 1-nitro-2-phenylpropene to NerA (0.73 mm) by a hyperbolic fit of the absorbance change at 524 nm.

In order to evaluate further our in silico analysis of the differences in the active sites between the OYEs compared above, we performed UV–visible absorbance difference titrations of NerA, OYE1, and XenA with 1-nitro-2-phenylpropene (Figure 4 A–D). According to the structure analysis (Figure 3) we expected that different binding modes should give rise to distinct perturbations of the UV–visible absorbance spectrum of the flavin cofactor. Indeed, we observed different UV–visible absorbance spectra for the enzymes at saturating substrate concentrations (Figure 4 A). The largest difference was seen between XenA and OYE1: with XenA the spectral perturbations were more pronounced and a strong charge-transfer absorbance at wavelengths >500 nm was observed. This is in contrast to OYE1, for which only minor perturbations were seen and the charge-transfer absorbance was virtually absent. This appears to reflect the (almost) exclusive nitro-reductase (XenA) and ene-reductase (OYE1) activities (Figure 4 A, solid and dashed lines).

In the case of NerA, the spectral perturbations appear to be a mixture of those observed with XenA and with OYE1: NerA shares the charge-transfer absorbance with XenA but the other features are more similar to OYE1. Again, this mirrors the dual catalytic activity of NerA as a nitro- and ene-reductase. Further studies with members of the OYE family are needed to substantiate the capability of UV/visible absorbance spectroscopy to predict the outcomes of substrate reduction by these enzymes.

## Conclusions

We determined the crystal structure of the enzyme NerA, which has been shown to exhibit dual ene-reductase and nitro-reductase activity with α,β-unsaturated nitro compounds, from *A. radiobacter*.^34^ NerA exhibits a TIM-barrel fold typical for OYE-like proteins, and all catalytically important active site residues are conserved. The loop β3 was found to be very long, similarly to that in the OYE from *S. oneidensis* (SYE1). The structure of NerA fits to previously proposed structural clusters and also exhibits sequence motifs that allowed prediction of the correct orientation of the residues Y65 and Y356, as well as its monomeric state. Soaking experiments resulted in two complex structures of the enzyme: one with 4-hydroxybenzaldehyde and one with 1-nitro-2-phenylpropene. Both compounds were found to exhibit typical OYE binding modes to NerA.

Apart from that, we used the structure of NerA to formulate a hypothesis for the nitro-reductase activity exhibited by a small subset of OYEs through comparison with the active sites of other OYE and OYE-like enzyme structures. Double bond reduction and nitro reduction could be the results of two competing binding modes of the nitro substrates. However, the ability to reduce nitro groups specifically is restricted to only a very limited set of OYEs, most likely owing to the presence of “hydrophobic hot-spots” in the region around loops β5 and β6.

## Experimental Section

**Reagents**: All chemicals were of highest grade commercially available and were purchased from Sigma–Aldrich, Fluka, or Merck. Nickel-nitrilotriacetic acid was from Qiagen.

**Cloning and expression in** ***E. coli***: A synthetic gene in *E. coli* codon usage, encoding for the GTN reductase from *A. radiobacter*, was ordered from GeneArt (Regensburg, Germany). It contained a C-terminal His_6_ tag for affinity purification, which was rendered excisable by introducing EagI restriction sites on both ends of the tag. The synthetic gene was flanked with NdeI and XhoI and was cloned into the NdeI/XhoI restriction sites of the pET21a vector (Novagen), to generate the expression plasmid pET21a(*NerA*). The sequence of the resulting expression plasmid was verified by DNA sequencing (Eurofins DNA, Ebersbach, Germany).

Chemically competent *E. coli* BL 21 (DE3) cells were transformed with pET21a(*NerA*), and protein expression was performed by standard protocols. Precultures were grown in lysogeny broth (LB) containing ampicillin (100 μg mL^−1^) at 37 °C for 16 h and were used to inoculate main cultures [800 mL LB medium containing ampicillin (100 μg mL^−1^)] to an initial OD_600_ of 0.05. Cells were grown at 37 °C to an OD_600_ of 0.6, and protein expression was induced by adding isopropyl-β-D-thiogalactopyranoside (IPTG) to a final concentration of 0.2 mm. The cultures were incubated at 37 °C for an additional 4 h, and cells were harvested by centrifugation. Cell pellets were washed with a NaCl solution (0.9 %, *w*/*v*) and stored at −20 °C.

**Cell disruption and purification**: The cell pellet was thawed in lysis buffer [phosphate buffer (50 mm) containing NaCl (300 mm) and imidazole (10 mm), pH 8.0], with use of 10 mL buffer per 5 g of wet cells. After addition of FMN (a few milligrams), cells were disrupted by sonication with cooling on ice. Cell debris was removed by centrifugation at 18 000 *g* for 30 min at 4 °C. The resulting supernatant was filtered through a 0.45 μm PVDF filter and was loaded onto a Ni-NTA column (Qiagen) equilibrated with lysis buffer. After loading of the filtered lysate, the column was washed with ten column volumes of wash buffer [phosphate buffer (50 mm) containing NaCl (300 mm) and imidazole (20 mm), pH 8.0]. Finally, the protein was eluted with elution buffer [phosphate buffer (50 mm) containing NaCl (300 mm) and imidazole (150 mm)], and fractions containing NerA (determined by SDS-PAGE) were pooled and dialyzed at 4 °C against Tris**⋅**HCl buffer (20 mm, pH 7.5) containing NaCl (150 mm). The resulting protein solution was concentrated to 30 mg mL^−1^ by using Amicon Ultra Centrifugal Filter Units with a molecular mass cut-off of 10 kDa (Millipore, Billerica, MA, USA). Aliquots (2 mL) of the concentrated protein solution were loaded onto a HiLoadTM 16/60 SuperdexTM 75 prep grade (Amersham Biosciences) gel filtration column equilibrated with Tris**⋅**HCl buffer (20 mm, pH 7.5) containing NaCl (150 mm), and were eluted at a flow rate of 1 mL min^−1^. The purities of the resulting fractions were determined by SDS-polyacrylamide gel electrophoresis, and fractions containing highly pure NerA were pooled and concentrated by ultrafiltration by use of the Amicon system as described above. Aliquots of concentrated protein with approximately 30 mg mL^−1^ were flash-frozen with nitrogen and stored at −20 °C.

**UV/Vis absorbance spectroscopy**: UV/Vis absorbance spectra were recorded with a Specord 210 spectrophotometer (Analytik Jena, Jena, Germany). Enzyme solutions for the titrations were taken from previously purified enzyme stock preparations. Difference titrations were performed for the enzymes NerA, XenA (≍30 μm), and OYE-1 (≍15 μm). Titrations were performed at room temperature in tandem cuvettes by the addition of solutions (1 mm and 10 mm) of 1-nitro-2-phenylpropene (dissolved in DMSO) to the corresponding enzyme solution (measurement cell) and to Tris**⋅**HCl buffer (50 mm, pH 8.0, reference cell). Recording intervals of 5 min were used. All acquired data were plotted by use of SigmaPlot.

**Crystallization**: After affinity and gel-filtration purification, native NerA was dialyzed overnight against Tris**⋅**HCl buffer (10 mm, pH 7.5). The dialyzed sample was concentrated by use of Centripreps (Millipore) to a final concentration of 26 mg mL^−1^. Several commercially available crystallization screens were set up by the microbatch technique with an Oryx 7 crystallization robot (Douglas Instruments Ltd). Crystallization drops contained equal amounts of protein and precipitant solution with final drop volumes of 1 μL. The setups were incubated both at 293 and 289 K. Initial crystallization trials yielded small, twinned, thin, needle-like crystals in various conditions. Optimization of the crystals was performed by matrix-seeding as described by D'Arcy;^41^ this yielded diffraction quality crystals under several sets of conditions: Hampton Index Screen F6 [ammonium sulfate (0.2 m), Bis**⋅**Tris (pH 6.0, 0.1 m), poly(ethylene glycol) 3350 (25 %, *w*/*v*)], Hampton Index Screen G3 [lithium sulfate monohydrate (0.2 m), Tris (pH 9.0, 0.1 m), poly(ethylene glycol) 3350 (25 %, *w*/*v*)], Hampton Index Screen H3 [sodium malonate (pH 7.0, 0.2 m), poly(ethylene glycol) 3350 (20 %, *w*/*v*)], Hampton PEG/Ion Screen D3 [sodium formate (pH 7.2, 0.2 m), poly(ethylene glycol) 3350 (20 %, *w*/*v*)]. For diffraction data collection, crystals were harvested from their mother liquor with CryoLoops (Hampton Research) and cryo-protected, prior to flash-cooling in liquid nitrogen and data collection, by soaking in a glycerol solution (25 %, *v*/*v*) for a few seconds.

**Inhibitor and substrate soaking experiments**: For the soaking of crystals with the inhibitor 4-hydroxybenzaldehyde, a solution (10 mm, 1.0 μL) was added to the drop and incubated for about three minutes. Subsequently the crystals were harvested and immediately flash-cooled in liquid nitrogen. For substrate soaking experiments, a solution of 1-nitro-2-phenylpropene (10 mm) in DMSO was prepared. Soakings were performed in a similar fashion as with the inhibitor, except that the soaking times were only 60 s, due to visible deterioration of the crystals after addition of the substrate solution (1 μL). The crystals were harvested from the solution and flash-cooled in liquid nitrogen.

**Structure determination**: Diffraction data were collected at beamline ID23-1 (*λ*=0.97240 Å) of the European Synchrotron Radiation Facility (ESRF) in Grenoble and at beamline X06DA-PXIII (*λ*=1.00 Å) of the Swiss Light Source (Paul Scherrer Institute, Villigen, Switzerland). The datasets were processed with the programs XDS and XSCALE^42^, ^43^ in the case of the apo dataset and with iMosflm,^44^ Scala,^45^ and programs of the CCP4 suite^46^ for the inhibitor and substrate soaks.

A BLAST search^47^ against the PDB revealed the structure of PETNR (PDB ID: 1H50) as the closest homologue to NerA (48 % sequence identity). The apo structure was determined by molecular replacement by use of the program PHASER^48^, ^49^ with the PETNR structure devoid of its cofactor as a search model. The resulting preliminary solution showed a high LLG value of 5572 and revealed three molecules to be present in the asymmetric unit, as was already anticipated due to a previously calculated Matthews coefficient of 2.3 Å^3^ Da^−1^ for the presence of three molecules.

Both complex structures were determined in a similar fashion, with use of the apo structure as a search model. However, in both complex structures the determined unit cell was smaller and contained one molecule per asymmetric unit. This was again in line with the calculated Matthews coefficient for these crystals.

**Structure refinement**: The initial model from the molecular replacement solution of the apo structure was further used as input for the automated chain-tracing/model building program PHENIX AutoBuild.^50^ The program was able to build an almost complete model into the electron density, resulting in *R*/*R*_free_ values of 21 and 24 %, respectively. This model was visually inspected with PyMOL (http://www.pymol.org) and showed chain A to be defined best. Chain A was therefore copied and superimposed onto chains B and C to give the best possible starting model for completion and refinement of the apo structure. Refinement against the high-resolution (1.6 Å) apo structure dataset, alternating with real-space fitting steps, with use of σ_A_-weighted 2*F*_o_−*F*_c_ and *F*_o_−*F*_c_ electron density maps, was performed with the programs PHENIX^50^ and COOT.^51^

For all datasets, *R*_free_ values^52^ were computed from 5 % randomly chosen reflections, which were not used during structure refinement. Water molecules were placed automatically into difference electron maps and accepted or rejected according to geometry criteria and their refined *B* factors. *B* factor refinement of individual sites was performed in later stages of the refinement. In the final refinement steps of the apo structure, a Translation-Libration-Skew (TLS) analysis was conducted with the aid of the TLS-MD web server (http://skuld.bmsc.washington.edu/∼tlsmd/)^53^, ^54^ and showed that four TLS groups per chain allow a sufficiently accurate description of the motions in the three chains. The TLS groups spanned residues 2–124, 125–135, 136–141, and 142–371 in chain A, residues 2–122, 123–141, 142–292, and 293–371 in chain B, and residues 2–124, 125–129, 130–141, and 142–371 in chain C. With these TLS groups as additional refinement parameters in PHENIX, both *R* and *R*_free_ values were decreased, yielding final values of *R*=15.87 % and *R*_free_=18.95 %. Structure validations were carried out with the web-based program MOLPROBITY,^55^ giving a Ramachandran plot with 97 % of the residues in favored, 2 % in allowed, and 1 % in disallowed regions for the apo structure. Residues with disallowed *Φ*/*Ψ* combinations were, however, well defined in the electron density.

The final structure of NerA in its apo form was used as the molecular replacement template for the lower-resolution (2.45 and 2.29 Å) complex structures. As in the case of the apo structure, refinement was performed with the programs PHENIX^50^ and COOT,^51^ with alternation of reciprocal space refinement with real-space fitting steps to σ_A_-weighted 2*F*_o_−*F*_c_ and *F*_o_−*F*_c_ electron density maps. TLS refinement was applied by use of PHENIX.^50^ Identification of TLS groups for the 4-hydroxybenzaldehyde and 1-nitro-2-phenylpropene soaking structures was conducted within PHENIX, with use of the option to find TLS groups automatically. This resulted in final structures with *R* values of *R*=19.43 % and *R*_free_=23.87 % for the 4-hydroxybenzaldehyde soak and *R*=21.35, *R*_free_=26.06 for the 1-nitro-2-phenylpropene structure. Validation of both complex structures was performed with MOLPROBITY,^55^ and this resulted in 97.6 % of all residues in favored, 2.1 % of all residues in allowed, and 0.3 % of all residues in disallowed regions for the 4-hydroxybenzaldehyde and 96.2 % in favored, 3.3 % in allowed, and 0.5 % in disallowed regions for the 1-nitro-2-phenylpropene structure. Details of the data collection, processing, and refinement of all three structures are summarized in Table 1. Structures and diffraction data have been deposited in the Protein Data Bank (http://rcsb.org/pdb) with the IDs 4JIC, 4JIP (complex with 4-hydroxybenzaldehyde), and 4JIQ (complex with 1-nitro-2-phenylpropene).

**Cavity analysis**: For the analysis of the active site cavities, structures of OYE enzymes were downloaded from the Protein Data Bank and examined with PyMol. The sizes and shapes of the active site cavities were calculated by using a LIGSITE algorithm.^56^ The hydrophobicity distributions of the cavities were analyzed with the hydrophobic calculation module of the program VASCo.^57^

## Supplementary material

As a service to our authors and readers, this journal provides supporting information supplied by the authors. Such materials are peer reviewed and may be re-organized for online delivery, but are not copy-edited or typeset. Technical support issues arising from supporting information (other than missing files) should be addressed to the authors.
