# Supplementary material for: The Structure of Glycerol Trinitrate Reductase NerA from Agrobacterium radiobacter Reveals the Molecular Reason for Nitro- and Ene-Reductase Activity in OYE Homologues
Source: Chembiochem. 2013 Apr 18;14(7):836–45. doi: 10.1002/cbic.201300136 (PMC3659409; doi:10.1002/cbic.201300136)
Supplement: Supplementary file 1 [file cbic0014-0836-SD1.pdf]

CHEMBIOCHEM

Supporting Information

© Copyright Wiley-VCH Verlag GmbH & Co. KGaA, 69451 Weinheim, 2013

The Structure of Glycerol Trinitrate Reductase NerA from *Agrobacterium radiobacter* Reveals the Molecular Reason for Nitro- and Ene-Reductase Activity in OYE Homologues

Gustav Oberdorfer,[a, b] Alexandra Binter,[a, c] Silvia Wallner,[c] Katharina Durchschein,[d]
Mélanie Hall,[d] Kurt Faber,[d] Peter Macheroux,[c] and Karl Gruber*[b]

cbic_201300136_sm_miscellaneous_information.pdf

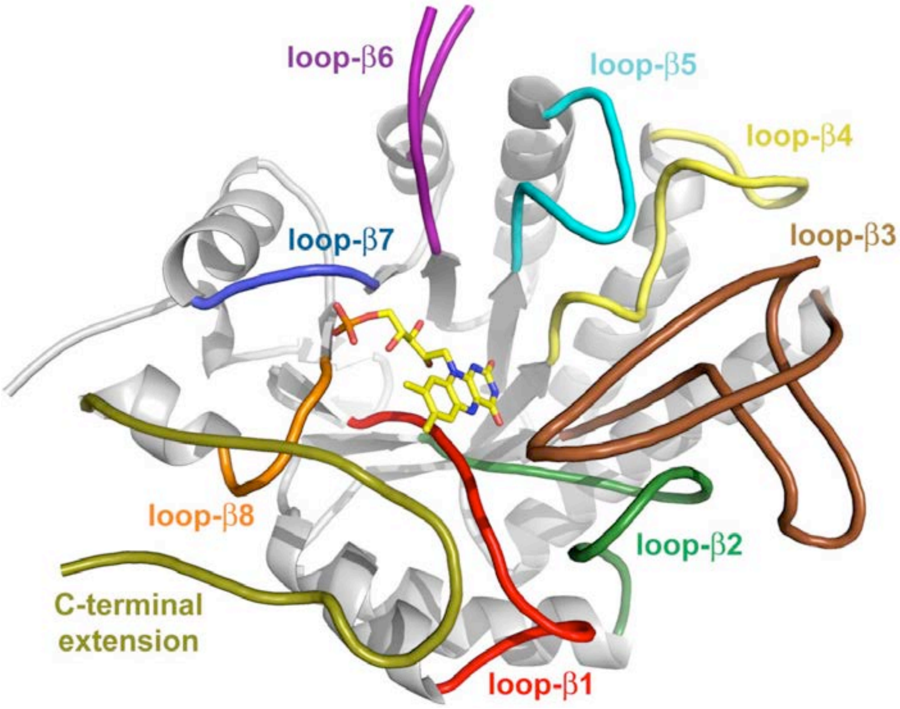

Supplementary Figure S1. Nomenclature of loops in OYE structures.

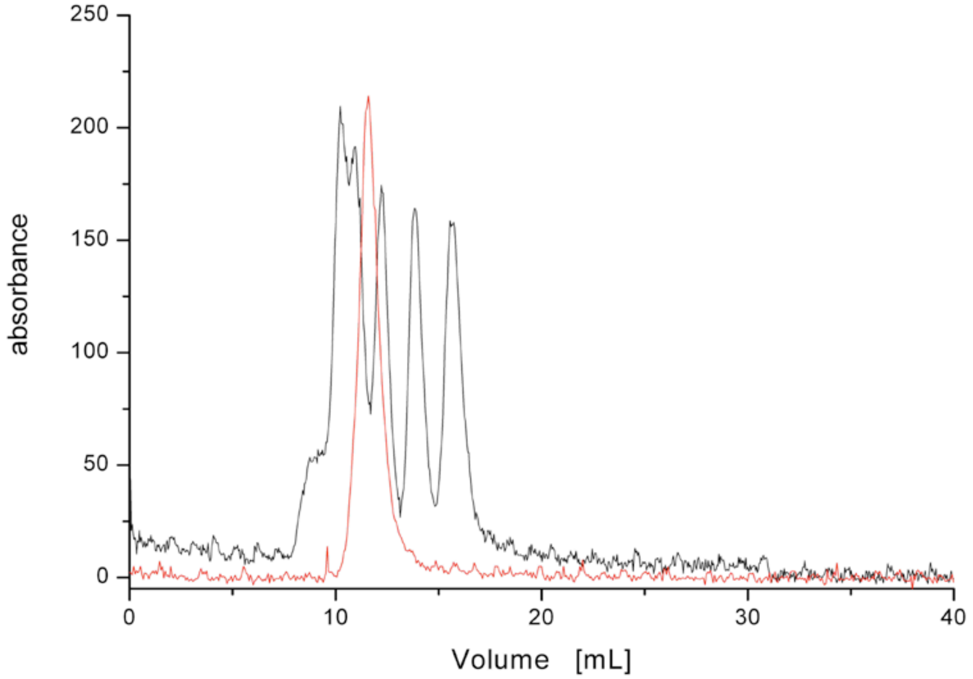

Supplementary Figure S2. Size exclusion chromatography of NerA using a Superdex™ 75 10/300 GL column (GE Heathcare) The red line shows the elution profile of NerA, whereas the black line shows the separation of a mix of calibration kit proteins (Aprotinin: 6500 Da, Ribonuclease A: 13700 Da; Carbonic anhydrase: 29000 Da; Ovalbumin: 44000 Da; Conalbumin: 75000 Da).
